# Neutralization Determinants on Poxviruses

**DOI:** 10.3390/v15122396

**Published:** 2023-12-08

**Authors:** Vernuccio Riccardo, Guardado-Calvo Pablo

**Affiliations:** Structural Biology of Infectious Diseases Unit, Institut Pasteur, Université Paris Cité, F-75015 Paris, France; riccardo.vernuccio@pasteur.fr

**Keywords:** poxvirus, monkeypox, mpox, vaccinia, neutralizing antibodies, vaccines, smallpox

## Abstract

Smallpox was a highly contagious disease caused by the variola virus. The disease affected millions of people over thousands of years and variola virus ranked as one of the deadliest viruses in human history. The complete eradication of smallpox in 1980, a major triumph in medicine, was achieved through a global vaccination campaign using a less virulent poxvirus, vaccinia virus. Despite this success, the herd immunity established by this campaign has significantly waned, and concerns are rising about the potential reintroduction of variola virus as a biological weapon or the emergence of zoonotic poxviruses. These fears were further fueled in 2022 by a global outbreak of monkeypox virus (mpox), which spread to over 100 countries, thereby boosting interest in developing new vaccines using molecular approaches. However, poxviruses are complex and creating modern vaccines against them is challenging. This review focuses on the structural biology of the six major neutralization determinants on poxviruses (D8, H3, A27, L1, B5, and A33), the localization of epitopes targeted by neutralizing antibodies, and their application in the development of subunit vaccines.

## 1. Introduction

Poxviruses (POXV) are a family of dsDNA viruses that replicate in the cytoplasm of infected cells; there are fourteen genera infecting vertebrates and four infecting insects. Members of the same genus are antigenically related and share similar morphology and host range. The genus orthopoxvirus (OPXV) is the best studied because it includes significant human pathogens such as variola (VARV) and monkeypox (MPOX) viruses and this review focuses on them. VARV is a remarkable virus for what it represents in the history of humanity and medicine. VARV is the etiological agent of smallpox, a highly contagious disease with a mortality rate exceeding 30% that killed hundreds of millions of people [1]. Individuals who recovered from smallpox, easily recognizable because of permanent scars, did not develop the disease again. This observation gave rise to a technique termed variolation, which consisted of blowing dried smallpox scabs into the nose of a healthy person who then developed a mild version of the disease and became immunized for life. In practice, around 1–2% of variolated people died. Variolation spread throughout the world and began to be used in Europe in the 18th century. In 1796, Edward Jenner, based on the principle of variolation, discovered vaccination. He essentially found that infection with a related, less virulent OPXV (cowpox virus) rendered individuals immune to smallpox [2]. This breakthrough paved the way for smallpox vaccination and the eradication of smallpox in the USA and Europe in the early 20th century. To achieve complete eradication, in 1959, the World Health Organization (WHO) launched the smallpox eradication programme using different strains of vaccinia virus (VACV) produced in the skin of animals: NYCBH (Dryvax^®^) in the USA, and Lister in the UK, to which we will refer later. The last natural case of smallpox was reported in 1977 and, in 1980, the disease was declared eradicated and the vaccination campaign suspended. The success of the vaccination programme can be attributed to three factors: vaccination generated long-term protection, VARV had no animal reservoirs besides humans, and vaccine-resistant strains did not emerge [3].

In the last years, there has been a growing interest in the development of new smallpox vaccines because herd immunity against OPXVs has waned over time and the current population is vulnerable to the deliberate reintroduction of VARV as a biological weapon or the emergence of zoonotic poxviruses such as MPOX, camelpox, or ORF virus [4]. A striking example is MPOX disease, a zoonotic disease endemic in central and western Africa caused by the MPOX virus. In 2022, a clade IIb (low-virulent) strain of MPOX adapted to human-to-human transmission caused a global outbreak that resulted in more than 90,000 cases and 157 deaths. This outbreak highlighted the real threat of poxvirus diseases and forced us to review what therapeutic tools we have at our disposal. First-generation smallpox vaccines are no longer available because of adverse effects, especially in individuals with immunodeficiencies or eczema [5], and because of the way they are produced, in the skin of live animals, they no longer meet the current safety standards. Second-generation vaccines addressed some of these issues by replacing live animals for virus production with tissue culture systems or embryonated chicken eggs. ACAM2000, approved by the FDA in 2007 and based on the NYCBH strain adapted to VERO cells, demonstrated similar immunogenicity as Dryvax^®^ but also caused severe adverse effects [6,7]. Efforts to improve the safety profile led to the development of the third-generation vaccines LC16m8 and MVA, which are attenuated viruses derived from the Lister and Ankara strains, respectively. Both have demonstrated safety and immunogenicity [8], but their effectiveness in preventing smallpox is unclear because they were developed after the eradication of the disease. In 2022, stockpile doses of MVA Bavarian Nordic (MVA-BN; also known as JYNNEOS^®^) were used to control MPOX disease. Subsequent studies found that two doses of MVA-BN resulted in low titers of MPOX-neutralizing antibodies compared with those observed in MPOX infection [9], and cases of vaccine breakthrough infections and reinfections were reported [10]. These findings raised concerns about vaccine effectiveness and have spurred interest in generating novel vaccines, either based on attenuated viruses modified to increase their immunogenicity or virulence or subunit vaccines using mRNA, DNA, or protein technologies [11,12,13,14,15,16].

## 2. The Replication Cycle of OPXVs

**Figure 1:**
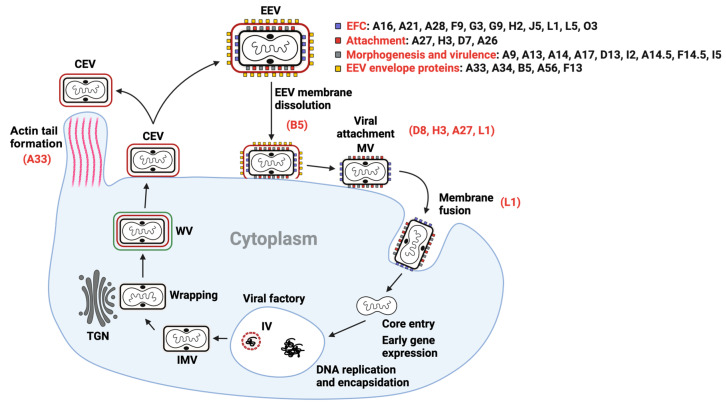
Schematic representation of the replication cycle of OPXVs. Above, a schematic of an enveloped particle (EV). The outer membrane is represented by a red line and the inner by a black line. The surface proteins of each membrane are indicated alongside. Below, the main steps of viral replication are indicated. The main neutralization determinants identified so far are highlighted in red, alongside the specific viral step they are involved in. Key abbreviations include MV for mature virion, IV for immature virion, IMV for intracellular mature virion, WV for wrapped virion, CEV for cell-associated virion, EEV for extracellular enveloped virion, and TGN for trans Golgi network. For more detailed information, please refer to the text.

Compared to most enveloped viruses, the structure and replication cycle of OPXVs is complex. The basic infectious form is the mature virion (MV), which is a large (360 × 270 × 250 nm) and brick-shaped particle composed of an outer membrane and a dumbbell-shaped core flanked by lateral bodies [17]. The outer membrane contains more than twenty surface proteins, comprising four responsible for attachment to target cells, eleven mediating fusion, and several associated with virus morphogenesis and virulence (Figure 1). The core contains a linear dsDNA genome of about 170–250 kbp encoding for more than 200 genes. MVs enter cells either through plasma or endocytic membrane fusion, a process mediated by the entry–fusion complex (EFC). Upon entry, the core is deposited in the cytoplasm and early proteins expressed. Then, the core dissolves, and the genome is released and surrounded by membranes and viral proteins to form a replication factory. Following DNA replication and intermediate/late gene expression, nascent virions are formed and the genome is encapsidated into immature virions (IV), which then mature into intracellular mature virions (IMV), the first infectious form of the virus. Most IMVs remain in the cytoplasm of infected cells until cell lysis occurs, then playing an important role in host-to-host transmissions. However, to spread within the host, some IMVs are covered by an additional double membrane to form wrapped virions (WV), which move to the periphery of cells to fuse with the plasma membrane and release EVs. Tecovirimat, the only drug licensed to treat smallpox and mpox blocks this wrapping step [18]. EVs that remain attached to the cell are called cell-associated enveloped viruses (CEV), those shedding from the cell extracellular enveloped viruses (EEV). Both EV forms are MVs wrapped with an additional membrane that contains seven EV-specific proteins: four transmembrane glycoproteins on the outer surface (A56, A33, A34, and B5), two proteins attached to A56 (K2 and C3), and one palmitoylated protein on the inner surface (F13). Deletion of any of the genes encoding for these proteins, except A56 and K2, results in decreased virus spread. B5 and A34 play a key role in virus entry, as they mediate the disruption of the outer membrane of EVs, exposing the fusion machinery on the MV [19].

## 3. Correlates of Protection

Developing subunit vaccines for complex pathogens requires a deep understanding of the molecular basis of protective immunity. This includes discerning the immune system branch that provides protection and determining the particular antigens and how many are responsible for eliciting it. Smallpox presents an extra challenge because the disease was eradicated before modern molecular techniques emerged and studies to understand protective immunity rely on related OPXVs, primarily MPOX and VACV [20,21,22,23]. Immune correlate analysis shows that while both immune system branches play a protective role, the presence of neutralizing antibodies (nAbs) provides the best correlation with protection [24,25,26], although neutralization in vitro does not always correlate with protection in vivo, as we will see below. Specifically, Edghill-Smith and colleagues showed, using macaques vaccinated with Dryvax [24], that depletion of B-cells, but not T-cells, abrogated vaccine-induced protection against MPOX and that the passive transfer of human antibodies protects non-immunized macaques from severe disease. Other studies showed that the level of preexisting antibodies in vaccinees inversely correlated with the rates of clinical symptoms associated with a new vaccination [27], and vaccinia immune globulin (VIG), a pool of antibodies collected from vaccinees, has been used to treat severe complications following smallpox vaccination [28].

## 4. Immunodominant Antigens

OPXVs encode for hundreds of proteins that can trigger an immune response, but not all are immunogenic. To pinpoint immunodominant antigens, Davies and colleagues developed a microarray assay printing VACV’s entire proteome and used it to profile sera from human and macaques vaccinated with MVA or Dryvax [29,30]. Others used a similar approach to analyze sera of non-human primates infected with VACV [31]. They found over twenty different antigens but only a handful present across most subjects. Surprisingly, most of these common antigens, such as D13, H5, A10, or A11, are not displayed on the surface of viral particles and presumably do not generate neutralizing antibodies. The role of these antibodies during infection remains unexplored but they may serve as decoys, diverting the immune system from targeting more critical proteins.

## 5. Neutralization Determinants

Not all immunodominant antigens generate nAbs, and not all neutralization determinants are immunodominant. For this reason, knowing how many and which of the surface antigens presented above mediate a neutralizing response is still a matter for discussion. Three main approaches have been used to identify and characterize neutralizing antibodies (nAbs) and their targets: neutralization tests, comet-reduction assays and antibody-depletion experiments. The conventional method for quantifying the ability of antibodies/sera to neutralize virus infection is the plaque-reduction neutralization test (PRNT). It quantifies the reduction in virus-induced plaques, with each plaque corresponding to one infectious virus particle. PRNT is the gold standard in the field and can be performed with any OPXV, allowing comparison of the neutralizing activity of a given antibody. However, it is time and labor-intensive, prone to counting errors, and unsuitable for large-scale screenings. To improve PRNT efficiency and reduce human error, alternative methods using VACV strains expressing reporter genes have been developed [32,33]. These are faster, more automated, and more reliable, but their use is limited to a small set of VACV strains modified to express reporter genes. Additionally, PRNT-independent techniques have emerged. Earl and colleagues developed a method [34] based on flow cytometry to count VACV-infected cells expressing GFP; they are fast and reliable but require specialized equipment and GFP-expressing VACV strains. Since OPXVs have two antigenically different particles (MV and EV), neutralization assays need to be adapted to each of them. These tests differ fundamentally in that, as EVs are fragile and easily release MVs, EV-neutralization assays require the presence of an MV-neutralizing antibody [35]. In both MV and EV-neutralization assays, the presence of the complement system is an important variable, since it enhances MV neutralization and is required to neutralize EVs. An alternative to measure EV neutralization that does not require an MV-neutralizing antibody is the so-called comet-reduction assay [36], which measures the ability of an antibody to reduce the size and number of the comet-shaped plaques formed by EVs released from an infected cell. These experiments are performed with the VACV IHDJ strain, which releases a higher amount of EVs [37,38]. In an antibody depletion assay [39], antibodies from a serum sample are mixed with a given antigen to form inactive antigen–antibody complexes. This assay allows for the understanding of the contribution of these “depleted” antibodies to the neutralizing capacity of the serum. Using these techniques, several neutralizing antigens have been identified. On the surface of MVs, these include H3 [21,35,40], D8 [35,41,42], A27 [35,39,43], L1 [35,44], A28 [45], and A17 [46], and on the surface of EVs, B5 [35,47,48] and A33 [35,49,50,51]. It is important to note that not all analyzed surface proteins induce a neutralizing response. Notably, mice immunized with plasmids coding for several EFC proteins (A16, A21, G3, G9, H2, J5, or L5), either alone or combined, fail to develop neutralizing antibodies [45]. This is unexpected, considering the EFC’s key role in viral entry (Figure 1) and the reasons for this lack of activity remain unclear. One possibility is that the conformation of isolated proteins differs from their structure in the EFC context, making them ineffective as immunogens. They might require be presented as stabilized complexes. Indeed, in many enveloped viruses, the most effective neutralizing antibodies target quaternary epitopes [52,53,54].

Multiple studies have shown that optimal protection is obtained with tetravalent vaccines combining two MV proteins (A27 and L1) and two EV antigens (B5 and A33) [55] or using a mixture of monoclonal antibodies targeting six surface proteins: D8, H3, A27, L1, B5, and A33 [35]. This review outlines the structural biology of these six proteins as well as the location of the neutralizing epitopes, where they are known. If the reader is interested in learning more about vaccines, correlates of protection, or antigenic determinants, we advise reading some of the excellent reviews that are available [3,16,56,57].

## 6. Neutralization Determinants on MVs

**Figure 2:**
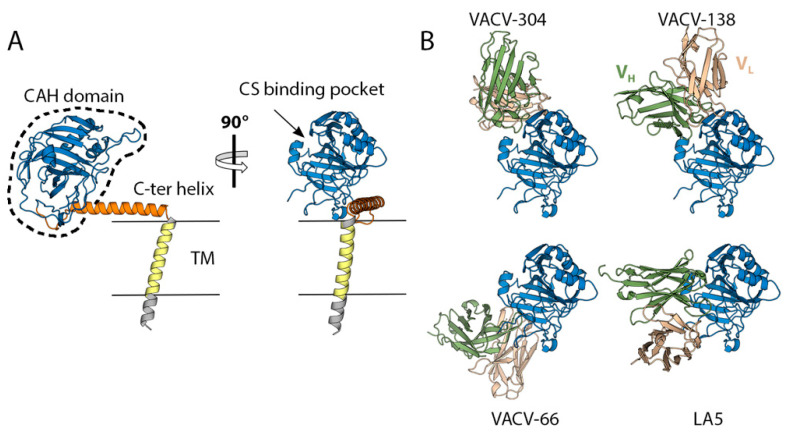
The structure of the chondroitin sulfate binding protein D8. (**A**) The structure of D8 obtained using AlphaFold2 [63] colored according to domains, as indicated. TM stands for transmembrane region. The region for which an experimental crystallographic model exists (PDB code: 4E9O, [64]) is indicated by a dashed line in the left panel. The right panel is an orthogonal view. (**B**) Crystallographic structures of the complexes formed by the CAH domain and the mAbs VACV-304 (PDB: 5USL, [62]), 138 (6B9J, [62]), 66 (5USH, [62]) and LA5 (4EBQ, [64]). The orientation of the CAH domain is the same as that shown in the right panel in A.

D8 is a membrane protein consisting of 304 amino acids (32 kDa, 93% sequence identity across pathogenic OPXVs) that binds to chondroitin sulfate E (CS-E) on the cell surface to provide virion attachment to target cells. D8 is not essential for virus propagation but is important for viral pathogenesis. VACV-ΔD8 (a VACV strain lacking D8 on its surface) replicates efficiently in tissue culture [42] but is 80% less lethal in mice [58]. D8 features a large, N-terminal globular domain with an inactive carbonic anhydrase fold (CAH, aa. 1–234), a C-terminal helix (aa. 235–273), a transmembrane segment (aa. 276–294), and a small C-terminal tail (Figure 2A). Functional studies indicate that the CAH domain binds CS-E through a conserved pocket and the C-terminal helix facilitates protein oligomerization [59].

Vaccination of mice with DNA coding for intact D8 or for a secreted, soluble version of D8 protects animals from dying but not from developing the disease [60]. Also, it does appear to considerably improve the protection of a tetravalent vaccine composed of A27, B5, L1, and A33. Multiple monoclonal antibodies (mAbs) have been isolated and characterized from various sources, including infected mice [61], macaques [31], and vaccinees and convalescents of mpox [35]. Some of them exhibit neutralizing activity in the presence of complement (Table 1). Biochemical and structural studies (Figure 2B) have classified these antibodies into two groups: those binding away from the CS-E pocket and those binding directly to or in close proximity to it, interfering with CS-E interaction [59]. Neutralization studies of antibodies belonging to both groups show that there is no link between the neutralizing activity and the ability to block interaction with CS-E [62], likely because the virus can utilize other GAG-binding proteins, such as H3 or A27, to adhere to target cells.

**Figure 3:**
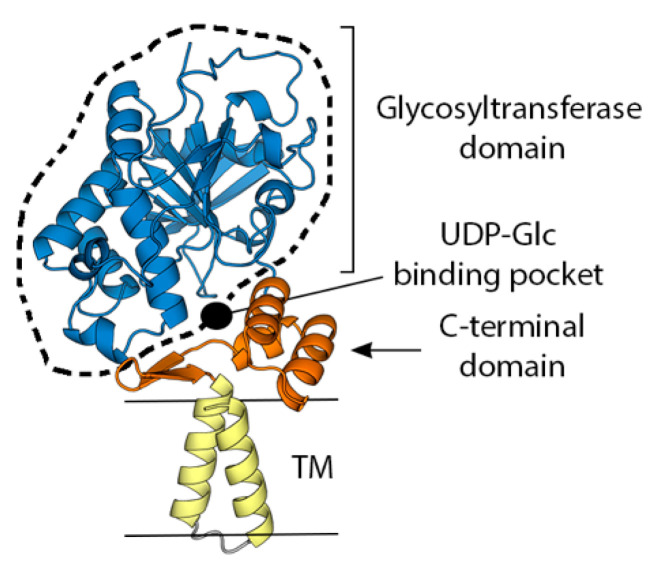
The structure of the heparan sulphate binding protein H3 obtained using AlphaFold2 [63]. The model is colored according to domains, as indicated. TM stands for transmembrane region. The region for which an experimental crystallographic model exists (5EJ0, [69]) is indicated by a dashed line.

H3 (also known as p35) is a membrane protein consisting of 324 amino acids (37 kDa, 93% sequence identity across pathogenic OPXVs) encoded by the gene H3L. Functionally, H3 is associated with virus attachment to the host cell, contributes to viral morphogenesis, and plays a crucial role in infection [68]. Electron microscopy images of cells infected with VACV-ΔH3 (a VACV strain lacking H3 expression) revealed impaired virion and decreased virus replication [68]. The atomic model of H3 (Figure 3) shows a large ectodomain formed by a globular domain with a glycosyltransferase fold (aa. 1–236), a smaller C-terminal domain (aa. 238–287), and two hydrophobic helices (aa. 289–304 and 310–324). These helices are either two transmembrane regions or a transmembrane region and an intravirion tail. Glycosyltransferases are enzymes that catalyze the transfer of an activated sugar to an acceptor molecule: a protein, a lipid, or a glycan. H3 specifically binds UDP–glucose and, akin to functional glycosyltransferases, features a conserved ExD motif that coordinates a divalent cation that is essential for UDP–glucose binding [69]. In the AF2 prediction, the sugar binding pocket is at the interface with the C-terminal domain, which is disordered in the crystal structure. This suggest that ligand binding may stabilize the interaction between the two domains (Figure 3). H3 binds in vitro and on the surface of mammalian cells heparan sulfate, probably through a positively charged surface. Protein–protein cross-linking mass spectrometry (XL–MS) studies on purified vaccinia virus show that H3 establishes multiple contacts with other MV surface proteins including A17, A27, and A26, with which it forms an attachment protein sub-network, and several contacts with EFC proteins, including A28, F9, G9, L5, and O3 [70].

H3 is an immunodominant and neutralizing antigen. Indeed, several antibody-profiling studies found H3 as the surface antigen that triggers the strongest response in most individuals [31,71,72]. Antibodies targeting H3, both polyclonal mixtures purified from VIG [21] and mAbs obtained from vaccinees [35] or macaques [31], show cross-neutralizing activity in the presence of complement and the presence of α-H3 Abs in serum showed a strong correlation with neutralizing activity [73]. Antibody depletion experiments performed with sera of individuals vaccinated with the Lister strain show that α-H3 antibodies account for up to 8% of MV-neutralizing activity [39]. Mice immunized with recombinant H3 produce high levels of neutralizing antibodies and were protected from a lethal intranasal challenge of the strain Western Reserve (WR) of VACV [21]. Similarly, mice receiving H3-neutralizing serum were also protected [21]. Interestingly, a mixture of human α-H3 neutralizing Abs does not appear to protect mice from VACV infection [35]. To date, the structure of any complex of a neutralizing antibody with H3 has not been reported, so the antigenic sites that induce a neutralizing response are not known.

**Figure 4:**
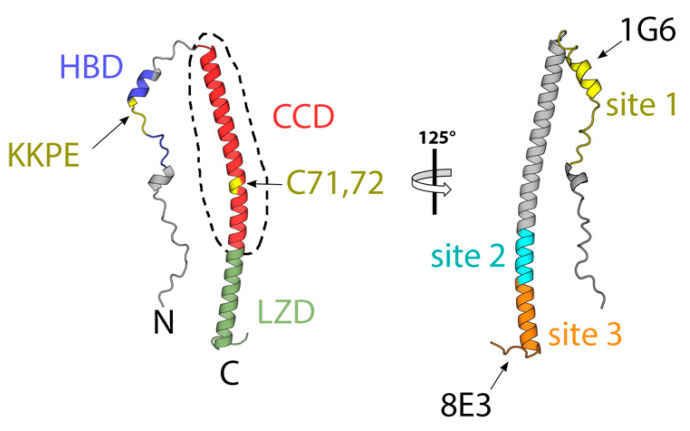
The structure of the heparan sulphate binding protein A27 obtained using AlphaFold2 [63]. The model is colored according to domains, as indicated. The region for which an experimental crystallographic model exists (3VOP, [74]) is indicated by a dashed line. The approximate locations of the epitopes of mAbs 1G6 (5EOQ, [65]) and 8E3 (5EOR, [65]) are indicated in the right panel.

A27 is a protein of 110 amino acids (12 kDa, 94% sequence identity across OPXVs) found on the surface of MV associated with the membrane protein A17. A27 binds heparan sulphate on the surface of host cells, promotes MV wrapping, and regulates the activity of the entry–fusion complex by recruiting A26 onto MV particles [74]. There is also some debate on whether A27 induces membrane fusion since the α-A27 mAb interferes with virus-induced cell–cell fusion [75] and co-expression of A27 and A17 triggers cell–cell fusion at an acidic pH [76]. The atomic model of A27, as illustrated in Figure 4, shows three functional regions: a heparin binding domain (HBD, aa. 21–34), which is essential for binding to cell surface; a coiled-coil domain (CCD, aa. 43–84), which is required for oligomerization in vivo and contains two cysteines (Cys71 and 72) that may form a disulfide bond with each other or with the cysteines 441 and 442 of A26 [74], and a leucine zipper domain (LZD, aa. 85–110), which binds A17 and anchors A27 to the MV surface. As mentioned above, in addition to the above-mentioned A17 and A26, XL–MS experiments suggest that A27 interacts with H3 [70].

Several studies have quantified titers of α-A27 in vaccinees and convalescents with varying outcomes. Sera from Dryvax, ACAM2000, or JYNNEOS vaccinees and VARV convalescents exhibit minimal α-A27 antibody levels [29,71]. However, sera from Lister vaccinees [39] and mpox convalescents display α-A27 seropositivity [77]. Serum depletion experiments using sera from a Lister vaccinee reveal that α-A27 antibodies account for up to 20% of MV-neutralizing activity [39], and a couple of mAbs isolated from a Dryvax vaccinee demonstrate MV-neutralizing activity against VACV, cowpox virus (CPXV), and MPOX [35]. These mAbs neutralize VACV MVs without complement, but complement presence enhances their activity by up to 100 times. In animal models, mice immunized with recombinant A27, while generating neutralizing antibodies [78], are not protected against a lethal intranasal VACV WR dose. Also, passive immunization of mice, either with rabbit α-A27 antibodies [78] or human nAbs [35], fails to confer protection against VACV. Studies utilizing mAbs from a mouse infected with a sub-lethal VACV dose identified four antigenic A27 sites [65]. Sites I, III, and IV are linear epitopes spanning residues 21–40, 81–100, and 91–110, respectively. Site II is a conformational epitope. mAbs targeting site I neutralize VACV in a complement-dependent manner, while mAbs binding to sites II, III, and IV are not neutralizing. Site I mAb 1G6 partially protects SCID mice infected with VACV ACAM2000, whereas site II and IV mAbs show limited beneficial effects.

**Figure 5:**
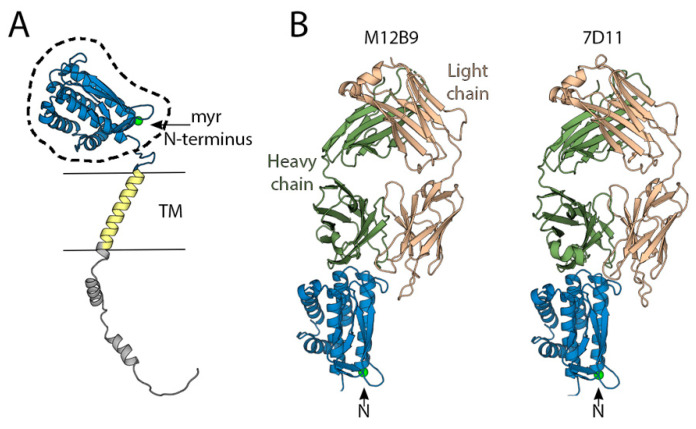
The structure of the MV surface protein L1 obtained using AlphaFold2 [63]. In the panel (**A**), the model is colored according to domains: the ectodomain in blue, the transmembrane (TM) segment in yellow, and the intraviral tail in gray. The region for which an experimental crystallographic model (1YPY, [83]) exists is indicated by a dashed line. Panel (**B**) shows the crystal structures of the complexes formed with the mAbs M12B9 (4U6H, [66]) and 7D11 (2I9L, [88]). The heavy and light chains are colored green and wheat, respectively. The N-terminus of L1 is indicated with a green dot and indicated with an arrow.

L1 is a myristoylated protein consisting of approximately 250 amino acids (27 kDa), which is highly conserved among OPXVs (98% sequence identity). It is present on the surface of mature virions (MVs) and is associated with the entry–fusion complex [79]. L1 plays a crucial role in several stages of viral entry, including membrane fusion and binding to target cells. α-L1 monoclonal antibodies have been shown to disrupt VACV cell–cell fusion at an acidic pH [80] and a recombinant, soluble form of L1 can bind to cells, inhibiting VACV entry into glycosaminoglycan (GAG)-deficient cells [81]. The deletion of the L1R gene blocks morphogenesis and prevents the production of infectious virus particles [82]. The atomic structure of L1 (Figure 5A), derived from a combination of crystallographic data [83] and an AF2 model, reveals three functional regions: a globular ectodomain (aa. 1–183), a TM region (aa. 184-204), and a large intraviral tail (aa. 205–250). The ectodomain is composed of a bundle of five helices and a pair of two-stranded β-sheets. The structure is stabilized by three conserved disulphide bonds formed in the reducing environment of the cytoplasm by a viral-encoded redox system [83]. Within infected cells, the N-terminus of L1 undergoes myristoylation, a post-translational modification (PTM) that is conserved across all poxviral L1 sequences. Adjacent to the N-terminus, there is a large hydrophobic cavity composed of 16 hydrophobic amino acids that is thought to host the myristoyl group. Mutations within this cavity have proven critical for infection [83]. Inhibition of N-myristoylation results in the production of non-infectious viruses that are defective in cell entry but not in replication or morphogenesis [84]. Based on the above, Foo and colleagues have proposed the “myristoyl switch model” [85], which suggests that L1 can exist in two states: an active and a resting state. In the resting state, the myristoyl group is sequestered within the hydrophobic pocket. Activation of the protein during viral entry produces a conformational change in the N-terminal helix, to which the myristoyl group is attached, leading to the exposure of the lipid group and its subsequent association with the cell membrane. Although this is a hypothetical model and there is no experimental evidence to support it, it could explain the neutralization mechanism of some mAbs, such as 7D11, which block core entry by binding to regions far from the N-terminal end, thereby preventing the transition to the active conformation of L1 (Figure 5B).

Mice immunized with a recombinant, soluble form of L1 produced in insect cells [36] or using an mRNA that encodes for a non-glycosylated form of M1 (the orthologue of L1 in MPOX) [14] induced a strong neutralizing response that partially protected mice from death after intranasal infection with VACV-WR but not from developing the disease, as attested by the weight loss of vaccinated animals. Similarly, macaques immunized with DNA encoding for L1R gene developed a severe disease but survived an MPOX infection [86]. In humans, several serological studies in vaccinated and convalescent patients show low or no antibody levels against L1 [35,39,71,87] but some mAbs isolated from vaccinees and mpox convalescent show complement-independent, cross-neutralizing activity against VACV, CPXV, and MPOX (Table 1). However, passive immunization with these nAbs did not protect mice from a lethal intranasal VACV challenge (31). Studies utilizing mAbs from a mouse infected with live VACV and boosted with a recombinant, non-glycosylated form of L1 identified three antigenic sites (I, II, and III). Only antibodies targeting site I neutralize VACV in a complement-independent manner and protect SCID mice infected with VACV-ACAM2000 [66]. Structural studies of site I mAb M12B9 reveal that it binds to four loops that connect the helix bundle at the membrane-distal region of L1 in an epitope that is very similar to that of the neutralizing antibody 7D11 (Figure 5B).

## 7. Neutralization Determinants on EVs

**Figure 6:**
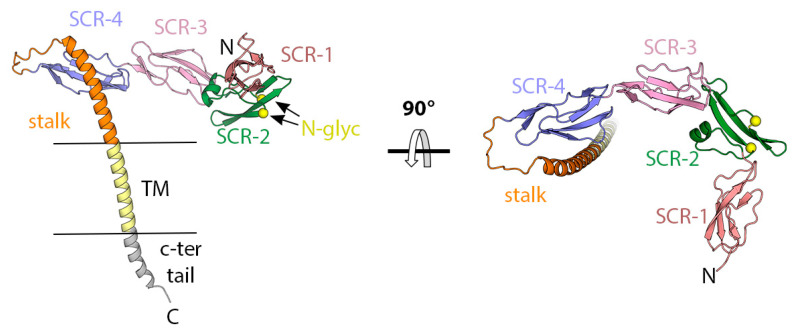
The structure of the EV surface glycoprotein B5 obtained using AlphaFold2 [63]. Two orthogonal views of the model colored according to domains, as indicated. TM stands for transmembrane region. The location of the N-glycosylation sequences in the SCR-2 domain are indicated with yellow spheres and labelled in the left panel.

B5 is a multifunctional type I membrane glycoprotein of about 317 amino acids (42 kDa, 93% sequence identity across OPXVs) found on the outer surface of EVs associated with A33, A34, and F13 [85,86]. During viral egress, B5 plays a crucial role, being required for the efficient wrapping of MVs [87] and for facilitating the transport of WVs to the plasma membrane [66]. Once outside, B5 interacts with A33/A36 to generate an actin tail. This results in a cellular protrusion with the virus at its tip, accelerating the spread of EVs to uninfected cells [88]. In viral entry, B5 and A34 mediate the non-fusogenic dissolution of the external membrane of EVs following interaction with GAGs [89]. There is no experimental structure of B5 available; however, sequence analysis and an AF2 prediction indicate that it is composed of four short-consensus repeat (SCR) domains (aa. 18–75, 76–128, 129–185, and 186–240), an acidic stalk region (aa. 241–279), a transmembrane region (aa. 280–300), and a short C-terminal tail (Figure 6). SCR domains are structural units characterized by a conserved CCCWC motif (two disulphide bonds and one Trp residue). They are present in many members of the complement control protein superfamily and play a pivotal role in the regulation of the complement system [90]. However, there is no evidence that B5 regulates complement activity. Several studies have explored the contribution of B5 and its domains to the formation of EVs and actin tails [91,92,93,94]. Modified VACV lacking B5 expression (VACV-ΔB5) secretes 10-fold fewer EVs than the wild type, resulting in small plaques in vitro and virus attenuation in vivo. Indeed, the vaccine strain LC16m8, derived from a small plaque isolate of VACV Lister [95], has a truncation of the B5R gene. Modified VACV strains lacking one or two of all SCRs produces more EVs than wild-type viruses and large plaques in vitro but is deficient in the induction of actin tails. This indicates that the SCR domains are dispensable for EV formation but required for the induction of actin bundles during virus egress. Analysis of viruses encoding a B5 protein lacking the C-terminal tail (VACV-ΔB5-CT) shows that the absence of this tail does not impact the formation of WVs, the development of actin tails, the production of EVs, or the size of virus plaques [94]. The stalk domain interacts with A34 and mediates the dissolution of the outer membrane [30,89]. Epitope mapping studies indicates that the stalk interacts with the first two SCRs domains creating important neutralization sites [35] but it is not clear whether this interaction is inter- or intramolecular.

In mice, injection of recombinant, soluble B5 (devoid of the TM segment) provided partial protection (30%) to mice challenged with an intranasal infection with VACV-WR [36,89] and passive immunization of mice with this serum, which has neutralizing activity, protects 40% of them from dying [89]. In humans, several studies confirmed the presence of α-B5 antibodies in the sera of vaccinees [29,30,35,71] and convalescents of smallpox [71] and mpox [35,87]. Human α-B5 antibodies neutralize EV infection in the presence of complement [35,90,91] and protects totally [91] or partially [35] animals challenged with VACV, depending on the strain and animal model used. Serum depletion experiments using sera from a Lister vaccinee show that B5 is the only target responsible for EV neutralization [39], and several mAbs isolated from Dryvax vaccinees and mpox convalescents demonstrate cross-neutralizing activity against VACV and CPXV [35]. Interestingly, despite some mAbs binding the MPOX ortholog of B5, none of them neutralize MPOX [35]. To date, the structure of any complex of a neutralizing antibody with B5 has not been reported, so the antigenic sites that induce a neutralizing response are not known.

**Figure 7:**
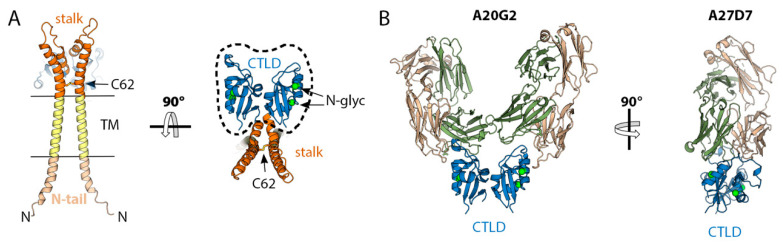
The structure of the EV surface glycoprotein A33. (**A**) structure of A33 obtained using AlphaFold2 [63] colored according to domains, as indicated. TM stands for transmembrane region. The left and right panels are orthogonal views, as indicated. The region for which an experimental crystallographic model exists (PDB code: 3K7B, [96]) is indicated by a dashed line. (**B**) Crystallographic structures of the complexes formed by the CTLD domain and the mAbs A20G2 (PDB: 4LU5, [67]), and A27D7 (4M1G, [67]). The orientation of the CTLD domain in the left panel is the same as that shown in the right panel in (**A**), the orientation in the right panel is rotated to optimize the view.

A33 is a type II membrane glycoprotein of about 185 amino acids (23–28 kDa, 94% sequence identity across OPXVs) found on the outer surface of EVs associated with B5 and A34 [92]. It is not essential for viral replication but it plays important roles in B5 trafficking [93], the formation of actin tails, and cell-to-cell spread of the virus [94]. VACV strains expressing truncated versions of A33 [95] or lacking A33 expression [94] released larger amounts of infectious particles to the medium but, as they cannot induce actin tails that project viral particles away from infected cells, they formed smaller plaques and are less virulent than the wild-type strain [94,95]. Biochemical studies of A33 from VACV show that it is a disulfide-bonded homodimer with two N-glycosylation sites at Asn 125 and 135 [49]. VARV and MPOX orthologs lacks the Asn-125 site. A model of the complete protein (Figure 7A), obtained by combining X-ray crystallography data [96] and an AF2 prediction, shows a short N-terminal intraviral region (aa. 1–33), a transmembrane segment (aa 34–56), and an ectodomain (aa. 57–185) formed by a flexible stalk (aa. 59–99) that contains an intermolecular disulphide bond and a globular homodimeric domain with a C-type lectin-like fold (CTLD, aa 100–185) similar to that found in dimeric NK-cell receptors [96].

In mice, vaccination with a recombinant, soluble form of A33 (devoid of the TM region, rA33), generates an immune response that protects 70% of animals from dying from intranasal infection with the WR strain of VACV [36,89] and passive immunization with the α-A33 mouse sera protects most animals [89]. Interestingly, as a unique immunogen, A33 is better than L1 or B5 but, unlike these, α-A33 sera have no neutralizing activity [89]. In humans, several studies have shown the presence of α-A33 antibodies in the sera of vaccinated and convalescent patients [29,30,35,71,87] and, as in mice, serum depletion experiments using sera from a Lister vaccine show that α-A33 antibodies have no neutralizing activity [39]. However, some mAbs isolated from mpox convalescent patients show complement-mediated, cross-neutralizing activity against VACV and MPOX (Table 1) and protect mice from a lethal VACV intranasal challenge [35]. To study the epitopes that mediate neutralization/protection, Matho and colleagues immunized a mouse with VACV followed by boosting with soluble rA33 [67]. They identified five nAbs (Table 1), and three of them (A2C7, A27D7, and A20G2) protected mice in vivo against disease (weight loss) and death [67]. They obtained the crystal structures of these three protective antibodies in complex with rA33 and showed that all bind the CTLD domain in a TM-distal epitope, which is presumably more exposed on the viral particle. The main difference is that A2C7 and A20G2 recognized a single protomer, i.e., there are two Fab molecules bound per rA33 dimer, and A27D7 is a quaternary antibody that recognizes a dimer of rA33 (Figure 7B). Unlike A2C7 and A20G2, A27D7 is quite resistant to epitope mutations and is able to bind rA33 of CPXV, MPOX, and ectromelia virus (ECTV) with picomolar affinities. A27D7 (but not A2C7 and A20G2) consistently protected mice challenged with ECTV [67].

## 8. Vaccines and Perspectives

The smallpox vaccine marked a significant milestone in medicine by eradicating smallpox. Its success is largely due to its ability to generate long-lasting, sterilizing immunity, setting a standard in vaccinology. However, smallpox vaccination was not safe for a substantial portion of the population, provoking the discontinuation of the vaccination campaign following the disease’s eradication. Currently, herd immunity established by vaccination is waning and the risks of poxvirus epidemics raising. A paradigmatic example is MPOX, a rodent-borne virus causing endemic zoonotic disease in Central (clade I) and West Africa (clade IIa). Although recurrent travel-related cases of MPOX had been reported outside Africa, in 2022 a large number of cases were reported globally. Genetic analyses identified a new clade (IIb), which has been circulating among humans since 2016 and exhibits molecular rates of evolution far exceeding those observed in clades I and IIa [97]. This discovery represents a pivotal change in the perception of the disease, shifting from a geographically restricted zoonosis to a globally prevalent human disease.

This paradigm shift spurred interest in the development of modern vaccines. They should be safe and cheap to reach a large number of people while providing broad, sterilizing and long-lasting immunity, similar to first-generation vaccines. Two main approaches are being followed: the generation of attenuated viruses and the development of subunit-based vaccines. Attenuated vaccines protect by targeting a large number of antigens. However, many immunodominant antigens do not elicit neutralizing antibodies and distract the immune system from targeting the crucial antigens and some neutralization determinants, such as L1 and A27, are poorly immunogenic. Also, these vaccines are difficult to produce in large scale. MVA-BN, a vaccine derived from an attenuated VACV strain, has been licensed by the FDA for the treatment of smallpox and MPOX diseases. It was used in 2022 to contain the MPOX pandemic. Retrospective studies confirmed that this vaccine is safe [98] but generates a low neutralizing response [9] that declines over time [99]. Fourth-generation attenuated vaccines are being developed to address these problems by deleting immunomodulatory genes, such as E3L or C6L, or reintroducing host-range genes like K1L or C7L [16].

Subunit-based vaccines address some of the issues mentioned above. They protect by targeting a select number of neutralizing antigens, they are safer and easier to produce on a large scale than attenuated viruses. However, as they elicit immunity against a limited number of antigens, the risk of the virus evolving to escape this immunity is higher. Overall, the effectiveness of these vaccines depends on the antigens and the technology used.

In terms of antigens, two aspects should be considered:

(1) The neutralization determinants and their biogenesis. To date, six well-characterized neutralization determinants have been identified: four on the membrane of MV (D8, A27, L1, and H3) and two on the EV membrane (B5 and A33). mRNA and DNA vaccines require antigens to either be secreted or presented on the plasma membrane. However, MV surface antigens do not naturally transit the secretory pathway and, thus, require some protein engineering to be used as immunogens in these platforms. Key modifications include adding a signal peptide to direct the protein to the secretory pathway—for instance, the signal peptide from influenza hemagglutinin was used to produce secreted M1 and A29 of MPOX [55]; the insertion, removal, or replacement of the transmembrane region; eliminating free cysteine residues, such as those found in A27 (Figure 4); and removing any N-glycosylation motifs. This last modification is particularly crucial in L1 (M1 in MPOX), as the epitope recognized by potent neutralizing antibodies contains a N-glycosylation motif. Additionally, surface antigens on EVs, which naturally transit to the plasma membrane, can also be modified to enhance their expression on the cell surface, for example by removing cytoplasmic regions or modifying their transmembrane domains [14];
(2) The number of antigens to be used. Immunizing mice with individual OPXV proteins may provide some protection against death, but in all cases, signs of disease are observed [14,36]. This is attributed to the complex nature of poxvirus infection, which results in the generation of two antigenically distinct viral particles: mature (MV) and enveloped (EV) virions, each playing distinct roles during infection. To achieve full protection, it is essential to neutralize both viral particles, which can only be accomplished by including MV and EV antigens. Following these guidelines, several subunit vaccines including between two and five antigens have demonstrated efficacy in protecting mice and nonhuman primates from lethal OPXV challenges (Table 2). The most recent mRNA vaccines, developed in response to the MPOX epidemics, use four antigens (the MPOX orthologs of A33, L1, B5, and A27), and not only confer complete survival to mice infected with VACV but also elicit higher neutralization titers than MVA and provide nearly total protection against disease [14,55].

## Figures and Tables

**Table 1:** List of selected neutralizing antibodies.

Antibody Name (PDB ^1^)	Species	Target and role in OPXV replication	VACV Neutralization IC50 ^2^ (Emax) ^3^		MPOX Neutralization IC50 (Emax)		Ref.
			−C ^4^	+C	−C	+C	
MV-neutralizing antibodies							
LA5 (4EBQ)	Mouse	D8 (attachment)	< ^5^	<10 (80)	ND ^6^	ND	[61]
VACV-304 (5USL)	Human		<	0.02 (79)	<	<	[35]
VACV-138 (6B9J)	Human		<	0.3 (80)	<	<	
VACV-66 (5USH)	Human		<	0.1 (85)	<	<	
VACV-249	Human		<	0.2 (70)	<	<	
MV-33	Macaque		<	<0.01 (90)	<	0.03 (60)	[31]
MV-49	Macaque		<	1 (>90)	ND	ND	
VACV-314	Human	H3 (attachment)	<	0.1 (74)	<	0.8 (84)	[35]
MPXV-72	Human		<	11.4 (66)	<	6.2 (64)	
MV-7	Macaque		<	2 (70)	ND	ND	[31]
MV-26	Macaque		<	0.1 (80)	ND	ND	
MV-31	Macaque		<	0.01 (80)	ND	ND	
MV-32	Macaque		<	0.06 (80)	<	1.0 (60)	
1G6 (5EOQ)	Mouse	A27 (attachment)	<	<20 (90)	ND	ND	[65]
VACV-301	Human		0.5 (61)	0.1 (77)	1.6 (84)	0.8 (92)	[35]
VACV-302	Human		12 (81)	0.1 (53)	0.1 (88)	6.3 (82)	
MPXV-26	Human	L1 (attachment, fusion)	0.3 (95)	0.7 (71)	3 (96)	6.2 (97)	[35]
M12B9 (4U6H)	Mouse		0.8 (100)	0.032 (100)	ND	ND	[66]
EV-neutralizing antibodies							
VACV-59	Human	B5 (spread, non-fusogenic EV membrane dissolution)	<	0.2 (72)	<	<	[35]
VACV-283	Human		<	0.7 (76)	<	<	
MPXV-13	Human		<	0.01 (80)	<	<	
MPXV-25	Human		<	0.02 (77)	<	<	
MPXV-51	Human	A33 (spread)	<	0.1 (50)	<	0.8 (77)	[35]
MPXV-56	Human		<	0.1 (56)	<	12.5 (75)	
A27D7 (4M1G)	Mouse		<	<10	ND	ND	[67]
A20G2 (4LU5)	Mouse		<	<10	ND	ND	
A2C7 (4LQF)	Mouse		<	<10	ND	ND	

^1^ Name of the antibody and PDB code of the complex when available, ^2^ IC_50_ = half maximal inhibitory concentration (μg/mL), ^3^ E_max_ = maximum level of neutralization achieved (%) by the antibody alone, ^4^ “C” (+ or −) indicates the presence or absence of complement in the neutralization test, ^5^ “<” indicates that no neutralization activity was detected, ^6^ ND = not determined.

**Table 2:** Subunit vaccines.

Vaccine Technology	MV Antigens	EV Antigens	Reference
DNA ^1^	L1	A33	[100]
Protein ^b,1^	A27	B5	[41]
Protein ^a,1^	L1	A33	[101]
Protein ^b,1^	A27, D8	B5	[41]
Protein ^c,1^	L1	A33, B5	[36]
Protein ^d,1^	L1	A33, B5	[102]
VEEV replicon ^2^	A27	A33, B5	[103]
mRNA ^3^	L1, A27	A33, B5	[14,15,55]
DNA ^1^	L1, A27	A33, B5	[86,104]
Protein ^d,1^	L1, A27	A33, B5	[105]
DNA ^3^	L1, A27	A33, B5	[106]
VEEV replicon ^1^	L1, A27	A33, B5	[107]
DNA ^1^	L1, A27, D8	A33, B5	[60]

Adjuvants used: ^a^ QS-21, ^b^ monophosphoryl lipid A + trehalose dicorynomycolate or TiterMax Gold as adjuvants, ^c^ saponin-type adjuvant, ^d^ CpG + aluminum hydroxide (alum); ^1^ VACV antigens; ^2^ cowpox virus antigens; ^3^ MPOX antigens.

## References

[1] Bray M., Buller M. (2004). Looking back at smallpox. Clin. Infect. Dis..

[2] Thurston L., Williams G. (2015). An examination of John Fewster’s role in the discovery of smallpox vaccination. J. R. Coll. Physicians Edinb..

[3] Moss B. (2011). Smallpox vaccines: Targets of protective immunity. Immunol. Rev..

[4] Yang Z., Gray M., Winter L. (2021). Why do poxviruses still matter?. Cell Biosci..

[5] Belongia E.A., Naleway A.L. (2003). Smallpox vaccine: The good, the bad, and the ugly. Clin. Med. Res..

[6] Weltzin R., Liu J., Pugachev K.V., Myers G.A., Coughlin B., Blum P.S., Nichols R., Johnson C., Cruz J., Kennedy J.S. (2003). Clonal vaccinia virus grown in cell culture as a new smallpox vaccine. Nat. Med..

[7] Monath T.P., Caldwell J.R., Mundt W., Fusco J., Johnson C.S., Buller M., Liu J., Gardner B., Downing G., Blum P.S. (2004). ACAM2000 clonal Vero cell culture vaccinia virus (New York City Board of Health strain)—A second-generation smallpox vaccine for biological defense. Int. J. Infect. Dis..

[8] Earl P.L., Americo J.L., Wyatt L.S., Eller L.A., Whitbeck J.C., Cohen G.H., Eisenberg R.J., Hartmann C.J., Jackson D.L., Kulesh D.A. (2004). Immunogenicity of a highly attenuated MVA smallpox vaccine and protection against monkeypox. Nature.

[9] Zaeck L.M., Lamers M.M., Verstrepen B.E., Bestebroer T.M., van Royen M.E., Gotz H., Shamier M.C., van Leeuwen L.P.M., Schmitz K.S., Alblas K. (2023). Low levels of monkeypox virus-neutralizing antibodies after MVA-BN vaccination in healthy individuals. Nat. Med..

[10] Hazra A., Zucker J., Bell E., Flores J., Gordon L., Mitja O., Suner C., Lemaignen A., Jamard S., Nozza S. (2023). Mpox in people with past infection or a complete vaccination course: A global case series. Lancet Infect. Dis..

[11] Xiao Y., Zeng Y., Schante C., Joshi S.B., Buchman G.W., Volkin D.B., Middaugh C.R., Isaacs S.N. (2020). Short-term and longer-term protective immune responses generated by subunit vaccination with smallpox A33, B5, L1 or A27 proteins adjuvanted with aluminum hydroxide and CpG in mice challenged with vaccinia virus. Vaccine.

[12] Golden J.W., Josleyn M., Mucker E.M., Hung C.F., Loudon P.T., Wu T.C., Hooper J.W. (2012). Side-by-side comparison of gene-based smallpox vaccine with MVA in nonhuman primates. PLoS ONE.

[13] Hou F., Zhang Y., Liu X., Murad Y.M., Xu J., Yu Z., Hua X., Song Y., Ding J., Huang H. (2023). mRNA vaccines encoding fusion proteins of monkeypox virus antigens protect mice from vaccinia virus challenge. Nat. Commun..

[14] Sang Y., Zhang Z., Liu F., Lu H., Yu C., Sun H., Long J., Cao Y., Mai J., Miao Y. (2023). Monkeypox virus quadrivalent mRNA vaccine induces immune response and protects against vaccinia virus. Signal Transduct. Target. Ther..

[15] Zhang R.R., Wang Z.J., Zhu Y.L., Tang W., Zhou C., Zhao S.Q., Wu M., Ming T., Deng Y.Q., Chen Q. (2023). Rational development of multicomponent mRNA vaccine candidates against mpox. Emerg. Microbes Infect..

[16] Sanchez-Sampedro L., Perdiguero B., Mejias-Perez E., Garcia-Arriaza J., Di Pilato M., Esteban M. (2015). The evolution of poxvirus vaccines. Viruses.

[17] Cyrklaff M., Risco C., Fernandez J.J., Jimenez M.V., Esteban M., Baumeister W., Carrascosa J.L. (2005). Cryo-electron tomography of vaccinia virus. Proc. Natl. Acad. Sci. USA.

[18] Jordan R., Leeds J.M., Tyavanagimatt S., Hruby D.E. (2010). Development of ST-246(R) for Treatment of Poxvirus Infections. Viruses.

[19] Law M., Carter G.C., Roberts K.L., Hollinshead M., Smith G.L. (2006). Ligand-induced and nonfusogenic dissolution of a viral membrane. Proc. Natl. Acad. Sci. USA.

[20] Slifka M.K. (2004). Immunological memory to viral infection. Curr. Opin. Immunol..

[21] Davies D.H., McCausland M.M., Valdez C., Huynh D., Hernandez J.E., Mu Y., Hirst S., Villarreal L., Felgner P.L., Crotty S. (2005). Vaccinia virus H3L envelope protein is a major target of neutralizing antibodies in humans and elicits protection against lethal challenge in mice. J. Virol..

[22] Law M., Putz M.M., Smith G.L. (2005). An investigation of the therapeutic value of vaccinia-immune IgG in a mouse pneumonia model. J. Gen. Virol..

[23] Lustig S., Fogg C., Whitbeck J.C., Eisenberg R.J., Cohen G.H., Moss B. (2005). Combinations of polyclonal or monoclonal antibodies to proteins of the outer membranes of the two infectious forms of vaccinia virus protect mice against a lethal respiratory challenge. J. Virol..

[24] Edghill-Smith Y., Golding H., Manischewitz J., King L.R., Scott D., Bray M., Nalca A., Hooper J.W., Whitehouse C.A., Schmitz J.E. (2005). Smallpox vaccine-induced antibodies are necessary and sufficient for protection against monkeypox virus. Nat. Med..

[25] Chaudhri G., Panchanathan V., Bluethmann H., Karupiah G. (2006). Obligatory requirement for antibody in recovery from a primary poxvirus infection. J. Virol..

[26] Xu R., Johnson A.J., Liggitt D., Bevan M.J. (2004). Cellular and humoral immunity against vaccinia virus infection of mice. J. Immunol..

[27] Orr N., Forman M., Marcus H., Lustig S., Paran N., Grotto I., Klement E., Yehezkelli Y., Robin G., Reuveny S. (2004). Clinical and immune responses after revaccination of israeli adults with the Lister strain of vaccinia virus. J. Infect. Dis..

[28] Kempe C.H. (1960). Studies smallpox and complications of smallpox vaccination. Pediatrics.

[29] Davies D.H., Wyatt L.S., Newman F.K., Earl P.L., Chun S., Hernandez J.E., Molina D.M., Hirst S., Moss B., Frey S.E. (2008). Antibody profiling by proteome microarray reveals the immunogenicity of the attenuated smallpox vaccine modified vaccinia virus ankara is comparable to that of Dryvax. J. Virol..

[30] Davies D.H., Liang X., Hernandez J.E., Randall A., Hirst S., Mu Y., Romero K.M., Nguyen T.T., Kalantari-Dehaghi M., Crotty S. (2005). Profiling the humoral immune response to infection by using proteome microarrays: High-throughput vaccine and diagnostic antigen discovery. Proc. Natl. Acad. Sci. USA.

[31] Noy-Porat T., Tamir H., Alcalay R., Rosenfeld R., Epstein E., Cherry L., Achdout H., Erez N., Politi B., Yahalom-Ronen Y. (2023). Generation of recombinant mAbs to vaccinia virus displaying high affinity and potent neutralization. Microbiol. Spectr..

[32] Manischewitz J., King L.R., Bleckwenn N.A., Shiloach J., Taffs R., Merchlinsky M., Eller N., Mikolajczyk M.G., Clanton D.J., Monath T. (2003). Development of a novel vaccinia-neutralization assay based on reporter-gene expression. J. Infect. Dis..

[33] Cosma A., Buhler S., Nagaraj R., Staib C., Hammarin A.L., Wahren B., Goebel F.D., Erfle V., Sutter G. (2004). Neutralization assay using a modified vaccinia virus Ankara vector expressing the green fluorescent protein is a high-throughput method to monitor the humoral immune response against vaccinia virus. Clin. Diagn. Lab. Immunol..

[34] Earl P.L., Americo J.L., Moss B. (2003). Development and use of a vaccinia virus neutralization assay based on flow cytometric detection of green fluorescent protein. J. Virol..

[35] Gilchuk I., Gilchuk P., Sapparapu G., Lampley R., Singh V., Kose N., Blum D.L., Hughes L.J., Satheshkumar P.S., Townsend M.B. (2016). Cross-Neutralizing and Protective Human Antibody Specificities to Poxvirus Infections. Cell.

[36] Fogg C., Lustig S., Whitbeck J.C., Eisenberg R.J., Cohen G.H., Moss B. (2004). Protective immunity to vaccinia virus induced by vaccination with multiple recombinant outer membrane proteins of intracellular and extracellular virions. J. Virol..

[37] Aldaz-Carroll L., Whitbeck J.C., Ponce de Leon M., Lou H., Hirao L., Isaacs S.N., Moss B., Eisenberg R.J., Cohen G.H. (2005). Epitope-mapping studies define two major neutralization sites on the vaccinia virus extracellular enveloped virus glycoprotein B5R. J. Virol..

[38] Law M., Hollinshead R., Smith G.L. (2002). Antibody-sensitive and antibody-resistant cell-to-cell spread by vaccinia virus: Role of the A33R protein in antibody-resistant spread. J. Gen. Virol..

[39] Putz M.M., Midgley C.M., Law M., Smith G.L. (2006). Quantification of antibody responses against multiple antigens of the two infectious forms of Vaccinia virus provides a benchmark for smallpox vaccination. Nat. Med..

[40] Lin C.L., Chung C.S., Heine H.G., Chang W. (2000). Vaccinia virus envelope H3L protein binds to cell surface heparan sulfate and is important for intracellular mature virion morphogenesis and virus infection in vitro and in vivo. J. Virol..

[41] Berhanu A., Wilson R.L., Kirkwood-Watts D.L., King D.S., Warren T.K., Lund S.A., Brown L.L., Krupkin A.K., Vandermay E., Weimers W. (2008). Vaccination of BALB/c mice with Escherichia coli-expressed vaccinia virus proteins A27L, B5R, and D8L protects mice from lethal vaccinia virus challenge. J. Virol..

[42] Niles E.G., Seto J. (1988). Vaccinia virus gene D8 encodes a virion transmembrane protein. J. Virol..

[43] Rodriguez J.F., Janeczko R., Esteban M. (1985). Isolation and characterization of neutralizing monoclonal antibodies to vaccinia virus. J. Virol..

[44] Wolffe E.J., Vijaya S., Moss B. (1995). A myristylated membrane protein encoded by the vaccinia virus L1R open reading frame is the target of potent neutralizing monoclonal antibodies. Virology.

[45] Shinoda K., Wyatt L.S., Moss B. (2010). The neutralizing antibody response to the vaccinia virus A28 protein is specifically enhanced by its association with the H2 protein. Virology.

[46] Wallengren K., Risco C., Krijnse-Locker J., Esteban M., Rodriguez D. (2001). The A17L gene product of vaccinia virus is exposed on the surface of IMV. Virology.

[47] Engelstad M., Howard S.T., Smith G.L. (1992). A constitutively expressed vaccinia gene encodes a 42-kDa glycoprotein related to complement control factors that forms part of the extracellular virus envelope. Virology.

[48] McCausland M.M., Benhnia M.R., Crickard L., Laudenslager J., Granger S.W., Tahara T., Kubo R., Koriazova L., Kato S., Crotty S. (2010). Combination therapy of vaccinia virus infection with human anti-H3 and anti-B5 monoclonal antibodies in a small animal model. Antivir. Ther..

[49] Roper R.L., Payne L.G., Moss B. (1996). Extracellular vaccinia virus envelope glycoprotein encoded by the A33R gene. J. Virol..

[50] Paran N., Lustig S., Zvi A., Erez N., Israely T., Melamed S., Politi B., Ben-Nathan D., Schneider P., Lachmi B. (2013). Active vaccination with vaccinia virus A33 protects mice against lethal vaccinia and ectromelia viruses but not against cowpoxvirus; elucidation of the specific adaptive immune response. Virol. J..

[51] Chen Z., Earl P., Americo J., Damon I., Smith S.K., Yu F., Sebrell A., Emerson S., Cohen G., Eisenberg R.J. (2007). Characterization of chimpanzee/human monoclonal antibodies to vaccinia virus A33 glycoprotein and its variola virus homolog in vitro and in a vaccinia virus mouse protection model. J. Virol..

[52] Mittler E., Serris A., Esterman E.S., Florez C., Polanco L.C., O’Brien C.M., Slough M.M., Tynell J., Groning R., Sun Y. (2023). Structural and mechanistic basis of neutralization by a pan-hantavirus protective antibody. Sci. Transl. Med..

[53] Mittler E., Wec A.Z., Tynell J., Guardado-Calvo P., Wigren-Bystrom J., Polanco L.C., O’Brien C.M., Slough M.M., Abelson D.M., Serris A. (2022). Human antibody recognizing a quaternary epitope in the Puumala virus glycoprotein provides broad protection against orthohantaviruses. Sci. Transl. Med..

[54] Rouvinski A., Guardado-Calvo P., Barba-Spaeth G., Duquerroy S., Vaney M.C., Kikuti C.M., Navarro Sanchez M.E., Dejnirattisai W., Wongwiwat W., Haouz A. (2015). Recognition determinants of broadly neutralizing human antibodies against dengue viruses. Nature.

[55] Freyn A.W., Atyeo C., Earl P.L., Americo J.L., Chuang G.Y., Natarajan H., Frey T.R., Gall J.G., Moliva J.I., Hunegnaw R. (2023). An mpox virus mRNA-lipid nanoparticle vaccine confers protection against lethal orthopoxviral challenge. Sci. Transl. Med..

[56] Kennedy R.B., Ovsyannikova I.G., Jacobson R.M., Poland G.A. (2009). The immunology of smallpox vaccines. Curr. Opin. Immunol..

[57] Meyer H., Ehmann R., Smith G.L. (2020). Smallpox in the Post-Eradication Era. Viruses.

[58] Rodriguez J.R., Rodriguez D., Esteban M. (1992). Insertional inactivation of the vaccinia virus 32-kilodalton gene is associated with attenuation in mice and reduction of viral gene expression in polarized epithelial cells. J. Virol..

[59] Matho M.H., de Val N., Miller G.M., Brown J., Schlossman A., Meng X., Crotty S., Peters B., Xiang Y., Hsieh-Wilson L.C. (2014). Murine anti-vaccinia virus D8 antibodies target different epitopes and differ in their ability to block D8 binding to CS-E. PLoS Pathog..

[60] Sakhatskyy P., Wang S., Chou T.H., Lu S. (2006). Immunogenicity and protection efficacy of monovalent and polyvalent poxvirus vaccines that include the D8 antigen. Virology.

[61] Meng X., Zhong Y., Embry A., Yan B., Lu S., Zhong G., Xiang Y. (2011). Generation and characterization of a large panel of murine monoclonal antibodies against vaccinia virus. Virology.

[62] Matho M.H., Schlossman A., Gilchuk I.M., Miller G., Mikulski Z., Hupfer M., Wang J., Bitra A., Meng X., Xiang Y. (2018). Structure-function characterization of three human antibodies targeting the vaccinia virus adhesion molecule D8. J. Biol. Chem..

[63] Jumper J., Evans R., Pritzel A., Green T., Figurnov M., Ronneberger O., Tunyasuvunakool K., Bates R., Zidek A., Potapenko A. (2021). Highly accurate protein structure prediction with AlphaFold. Nature.

[64] Matho M.H., Maybeno M., Benhnia M.R., Becker D., Meng X., Xiang Y., Crotty S., Peters B., Zajonc D.M. (2012). Structural and biochemical characterization of the vaccinia virus envelope protein D8 and its recognition by the antibody LA5. J. Virol..

[65] Kaever T., Matho M.H., Meng X., Crickard L., Schlossman A., Xiang Y., Crotty S., Peters B., Zajonc D.M. (2016). Linear Epitopes in Vaccinia Virus A27 Are Targets of Protective Antibodies Induced by Vaccination against Smallpox. J. Virol..

[66] Kaever T., Meng X., Matho M.H., Schlossman A., Li S., Sela-Culang I., Ofran Y., Buller M., Crump R.W., Parker S. (2014). Potent neutralization of vaccinia virus by divergent murine antibodies targeting a common site of vulnerability in L1 protein. J. Virol..

[67] Matho M.H., Schlossman A., Meng X., Benhnia M.R., Kaever T., Buller M., Doronin K., Parker S., Peters B., Crotty S. (2015). Structural and Functional Characterization of Anti-A33 Antibodies Reveal a Potent Cross-Species Orthopoxviruses Neutralizer. PLoS Pathog..

[68] da Fonseca F.G., Wolffe E.J., Weisberg A., Moss B. (2000). Effects of deletion or stringent repression of the H3L envelope gene on vaccinia virus replication. J. Virol..

[69] Singh K., Gittis A.G., Gitti R.K., Ostazeski S.A., Su H.P., Garboczi D.N. (2016). The Vaccinia Virus H3 Envelope Protein, a Major Target of Neutralizing Antibodies, Exhibits a Glycosyltransferase Fold and Binds UDP-Glucose. J. Virol..

[70] Mirzakhanyan Y., Gershon P. (2019). The Vaccinia virion: Filling the gap between atomic and ultrastructure. PLoS Pathog..

[71] Davies D.H., Molina D.M., Wrammert J., Miller J., Hirst S., Mu Y., Pablo J., Unal B., Nakajima-Sasaki R., Liang X. (2007). Proteome-wide analysis of the serological response to vaccinia and smallpox. Proteomics.

[72] Townsend M.B., Keckler M.S., Patel N., Davies D.H., Felgner P., Damon I.K., Karem K.L. (2013). Humoral immunity to smallpox vaccines and monkeypox virus challenge: Proteomic assessment and clinical correlations. J. Virol..

[73] Benhnia M.R., McCausland M.M., Su H.P., Singh K., Hoffmann J., Davies D.H., Felgner P.L., Head S., Sette A., Garboczi D.N. (2008). Redundancy and plasticity of neutralizing antibody responses are cornerstone attributes of the human immune response to the smallpox vaccine. J. Virol..

[74] Chang T.H., Chang S.J., Hsieh F.L., Ko T.P., Lin C.T., Ho M.R., Wang I., Hsu S.T., Guo R.T., Chang W. (2013). Crystal structure of vaccinia viral A27 protein reveals a novel structure critical for its function and complex formation with A26 protein. PLoS Pathog..

[75] Rodriguez J.F., Paez E., Esteban M. (1987). A 14,000-Mr envelope protein of vaccinia virus is involved in cell fusion and forms covalently linked trimers. J. Virol..

[76] Kochan G., Escors D., Gonzalez J.M., Casasnovas J.M., Esteban M. (2008). Membrane cell fusion activity of the vaccinia virus A17-A27 protein complex. Cell Microbiol..

[77] Cohn H., Bloom N., Cai G.Y., Clark J.J., Tarke A., Bermudez-Gonzalez M.C., Altman D.R., Lugo L.A., Lobo F.P., Marquez S. (2023). Mpox vaccine and infection-driven human immune signatures: An immunological analysis of an observational study. Lancet Infect. Dis..

[78] Fogg C.N., Americo J.L., Earl P.L., Resch W., Aldaz-Carroll L., Eisenberg R.J., Cohen G.H., Moss B. (2008). Disparity between levels of in vitro neutralization of vaccinia virus by antibody to the A27 protein and protection of mice against intranasal challenge. J. Virol..

[79] Schin A.M., Diesterbeck U.S., Moss B. (2021). Insights into the Organization of the Poxvirus Multicomponent Entry-Fusion Complex from Proximity Analyses in Living Infected Cells. J. Virol..

[80] Ichihashi Y., Takahashi T., Oie M. (1994). Identification of a vaccinia virus penetration protein. Virology.

[81] Foo C.H., Lou H., Whitbeck J.C., Ponce-de-Leon M., Atanasiu D., Eisenberg R.J., Cohen G.H. (2009). Vaccinia virus L1 binds to cell surfaces and blocks virus entry independently of glycosaminoglycans. Virology.

[82] Ravanello M.P., Hruby D.E. (1994). Conditional lethal expression of the vaccinia virus L1R myristylated protein reveals a role in virion assembly. J. Virol..

[83] Su H.P., Garman S.C., Allison T.J., Fogg C., Moss B., Garboczi D.N. (2005). The 1.51-Angstrom structure of the poxvirus L1 protein, a target of potent neutralizing antibodies. Proc. Natl. Acad. Sci. USA.

[84] Priyamvada L., Kallemeijn W.W., Faronato M., Wilkins K., Goldsmith C.S., Cotter C.A., Ojeda S., Solari R., Moss B., Tate E.W. (2022). Inhibition of vaccinia virus L1 N-myristoylation by the host N-myristoyltransferase inhibitor IMP-1088 generates non-infectious virions defective in cell entry. PLoS Pathog..

[85] Foo C.H., Whitbeck J.C., Ponce-de-Leon M., Saw W.T., Cohen G.H., Eisenberg R.J. (2012). The myristate moiety and amino terminus of vaccinia virus l1 constitute a bipartite functional region needed for entry. J. Virol..

[86] Hooper J.W., Thompson E., Wilhelmsen C., Zimmerman M., Ichou M.A., Steffen S.E., Schmaljohn C.S., Schmaljohn A.L., Jahrling P.B. (2004). Smallpox DNA vaccine protects nonhuman primates against lethal monkeypox. J. Virol..

[87] Otter A.D., Jones S., Hicks B., Bailey D., Callaby H., Houlihan C., Rampling T., Gordon N.C., Selman H., Satheshkumar P.S. (2023). Monkeypox virus-infected individuals mount comparable humoral immune responses as Smallpox-vaccinated individuals. Nat. Commun..

[88] Su H.P., Golden J.W., Gittis A.G., Hooper J.W., Garboczi D.N. (2007). Structural basis for the binding of the neutralizing antibody, 7D11, to the poxvirus L1 protein. Virology.

[89] Galmiche M.C., Goenaga J., Wittek R., Rindisbacher L. (1999). Neutralizing and protective antibodies directed against vaccinia virus envelope antigens. Virology.

[90] Aldaz-Carroll L., Xiao Y., Whitbeck J.C., de Leon M.P., Lou H., Kim M., Yu J., Reinherz E.L., Isaacs S.N., Eisenberg R.J. (2007). Major neutralizing sites on vaccinia virus glycoprotein B5 are exposed differently on variola virus ortholog B6. J. Virol..

[91] Paran N., Lustig S. (2010). Complement-bound human antibodies to vaccinia virus B5 antigen protect mice from virus challenge. Expert Rev. Vaccines.

[92] Monticelli S.R., Earley A.K., Stone R., Norbury C.C., Ward B.M. (2020). Vaccinia Virus Glycoproteins A33, A34, and B5 Form a Complex for Efficient Endoplasmic Reticulum to trans-Golgi Network Transport. J. Virol..

[93] Chan W.M., Ward B.M. (2010). There is an A33-dependent mechanism for the incorporation of B5-GFP into vaccinia virus extracellular enveloped virions. Virology.

[94] Roper R.L., Wolffe E.J., Weisberg A., Moss B. (1998). The envelope protein encoded by the A33R gene is required for formation of actin-containing microvilli and efficient cell-to-cell spread of vaccinia virus. J. Virol..

[95] Katz E., Wolffe E., Moss B. (2002). Identification of second-site mutations that enhance release and spread of vaccinia virus. J. Virol..

[96] Su H.P., Singh K., Gittis A.G., Garboczi D.N. (2010). The structure of the poxvirus A33 protein reveals a dimer of unique C-type lectin-like domains. J. Virol..

[97] O’Toole A., Neher R.A., Ndodo N., Borges V., Gannon B., Gomes J.P., Groves N., King D.J., Maloney D., Lemey P. (2023). APOBEC3 deaminase editing in mpox virus as evidence for sustained human transmission since at least 2016. Science.

[98] Duffy J., Marquez P., Moro P., Weintraub E., Yu Y., Boersma P., Donahue J.G., Glanz J.M., Goddard K., Hambidge S.J. (2022). Safety Monitoring of JYNNEOS Vaccine During the 2022 Mpox Outbreak—United States, May 22–October 21, 2022. MMWR Morb. Mortal. Wkly Rep..

[99] Priyamvada L., Carson W.C., Ortega E., Navarra T., Tran S., Smith T.G., Pukuta E., Muyamuna E., Kabamba J., Nguete B.U. (2022). Serological responses to the MVA-based JYNNEOS monkeypox vaccine in a cohort of participants from the Democratic Republic of Congo. Vaccine.

[100] Hooper J.W., Custer D.M., Schmaljohn C.S., Schmaljohn A.L. (2000). DNA vaccination with vaccinia virus L1R and A33R genes protects mice against a lethal poxvirus challenge. Virology.

[101] Fogg C.N., Americo J.L., Lustig S., Huggins J.W., Smith S.K., Damon I., Resch W., Earl P.L., Klinman D.M., Moss B. (2007). Adjuvant-enhanced antibody responses to recombinant proteins correlates with protection of mice and monkeys to orthopoxvirus challenges. Vaccine.

[102] Xiao Y., Aldaz-Carroll L., Ortiz A.M., Whitbeck J.C., Alexander E., Lou H., Davis H.L., Braciale T.J., Eisenberg R.J., Cohen G.H. (2007). A protein-based smallpox vaccine protects mice from vaccinia and ectromelia virus challenges when given as a prime and single boost. Vaccine.

[103] Thornburg N.J., Ray C.A., Collier M.L., Liao H.X., Pickup D.J., Johnston R.E. (2007). Vaccination with Venezuelan equine encephalitis replicons encoding cowpox virus structural proteins protects mice from intranasal cowpox virus challenge. Virology.

[104] Hooper J.W., Custer D.M., Thompson E. (2003). Four-gene-combination DNA vaccine protects mice against a lethal vaccinia virus challenge and elicits appropriate antibody responses in nonhuman primates. Virology.

[105] Buchman G.W., Cohen M.E., Xiao Y., Richardson-Harman N., Silvera P., DeTolla L.J., Davis H.L., Eisenberg R.J., Cohen G.H., Isaacs S.N. (2010). A protein-based smallpox vaccine protects non-human primates from a lethal monkeypox virus challenge. Vaccine.

[106] Heraud J.M., Edghill-Smith Y., Ayala V., Kalisz I., Parrino J., Kalyanaraman V.S., Manischewitz J., King L.R., Hryniewicz A., Trindade C.J. (2006). Subunit recombinant vaccine protects against monkeypox. J. Immunol..

[107] Hooper J.W., Ferro A.M., Golden J.W., Silvera P., Dudek J., Alterson K., Custer M., Rivers B., Morris J., Owens G. (2009). Molecular smallpox vaccine delivered by alphavirus replicons elicits protective immunity in mice and non-human primates. Vaccine.

